# Supplementary figures and images for: Machine learning and dengue forecasting: Comparing random forests and artificial neural networks for predicting dengue burden at national and sub-national scales in Colombia
Source: PLoS Negl Trop Dis. 2020 Sep 24;14(9):e0008056. doi: 10.1371/journal.pntd.0008056 (PMC7537891; doi:10.1371/journal.pntd.0008056)

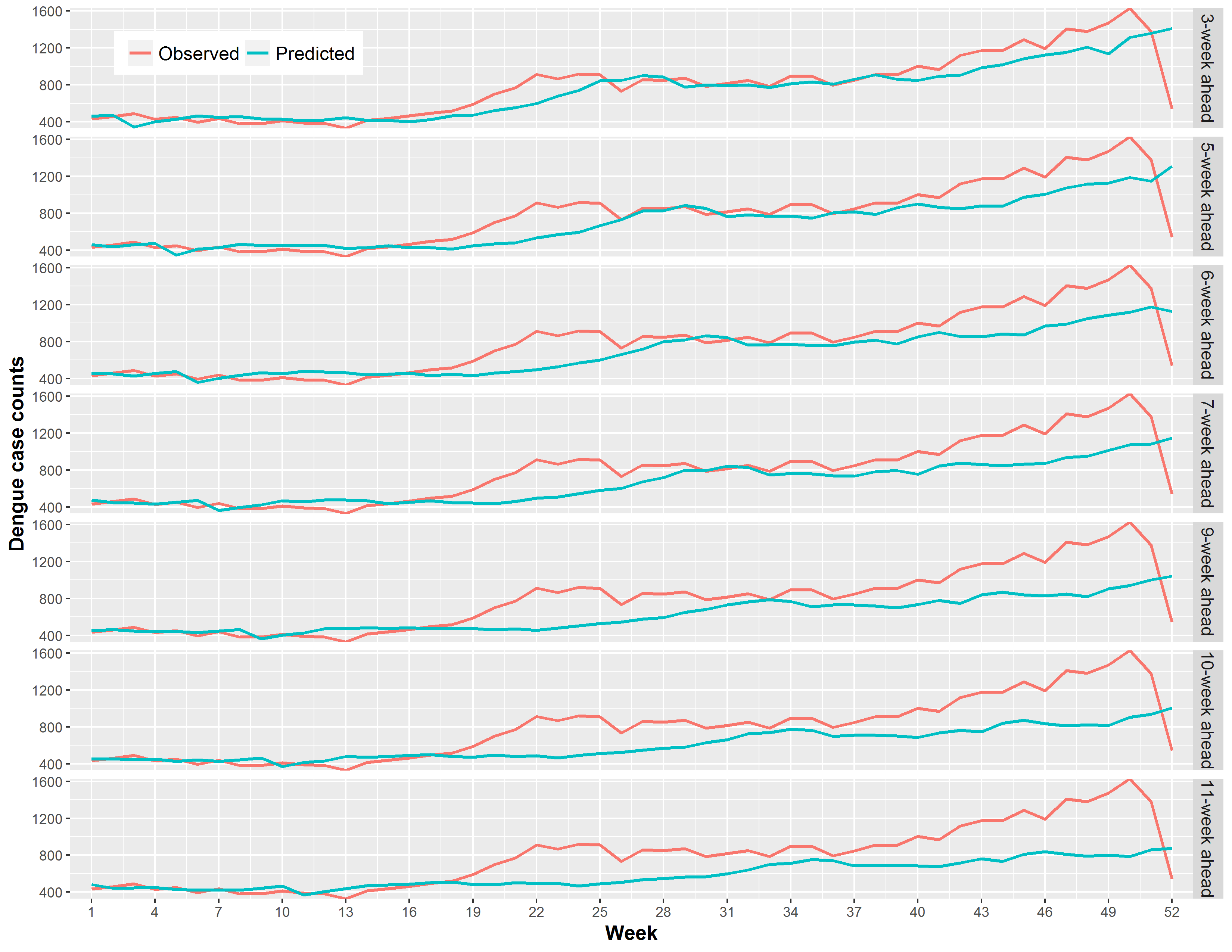

Supplement: S1 Fig — (TIFF) [file pntd.0008056.s001.tiff]

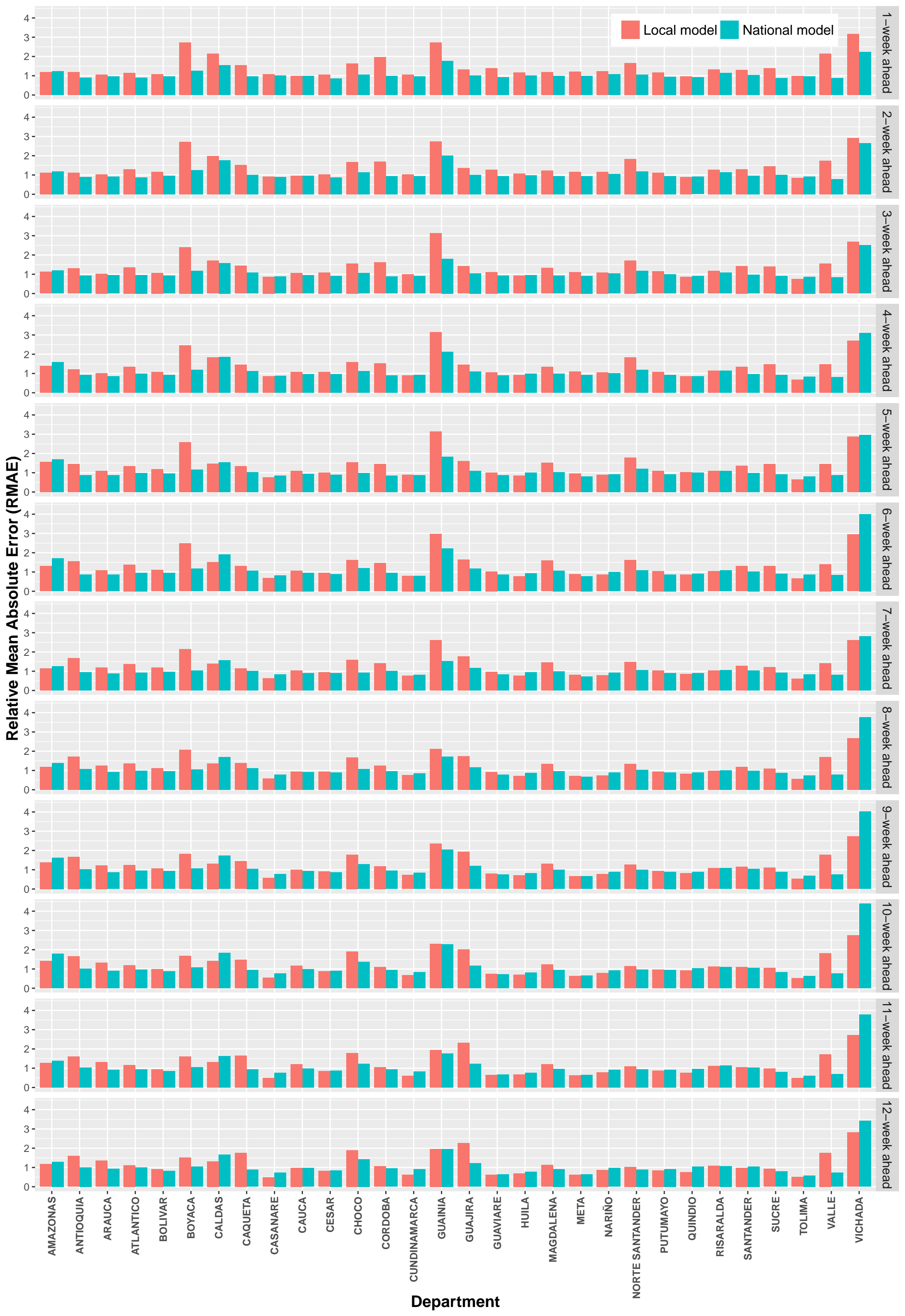

Supplement: S2 Fig — (PDF) [file pntd.0008056.s002.pdf]
