# Supplementary material for: Machine learning and dengue forecasting: Comparing random forests and artificial neural networks for predicting dengue burden at national and sub-national scales in Colombia
Source: PLoS Negl Trop Dis. 2020 Sep 24;14(9):e0008056. doi: 10.1371/journal.pntd.0008056 (PMC7537891; doi:10.1371/journal.pntd.0008056)
Supplement: Attachment — Submitted filename: response_letter_final.docx [file pntd.0008056.s003.docx]

Dear Dr. Choisy, Dr. Reiner, and Reviewers:

Thank you for your comments concerning our revised manuscript, “Machine learning and dengue forecasting: Comparing random forests and artificial neural networks for predicting dengue burdens at the national sub-national scale in Colombia (PNTD-D-19-02179).” The comments are very valuable and helpful in improving our paper. We have studied the comments carefully and made revisions that we hope will meet with your approval. Please note that we have included Table 5 at the end of the manuscript given its landscape format and also that we would like to acknowledge the reviewers (in the Acknowledgement section). The main revisions in the manuscript and responses to the reviewers’ comments are as follows:

**Reviewer 1**

1. The study design needs an appropriate baseline model to truly address the stated objective. In the introduction, the authors state that "conventional time-series models such as ARIMA are insufficient to meet complex forecasting requirements", however that is only known because they are compared to baseline models that are commonly used by public officials. In a way, the authors use the Local Model as the baseline, but we have no reason to trust this as a baseline model because we would like to evaluate this model as well. For predictions of Dengue_{k+1}, a good naive baseline model would be Dengue_k because public health officials often react to the most recent data point. For longer term predictions, it would be good to show how the RFs perform relative to ARIMA models. A paper that describes selecting a baseline model for dengue prediction in detail is Reich et al., 2015, Case Study in Evaluating Time Series Prediction Models Using the Relative Mean Absolute Error.

*Response*: We appreciate the reviewer’s comment and have added ARIMA baseline models: [lines 220-226]: Standard univariate ARIMA developed at the local scale was used as the baseline to compare with the RF and ANN models. The Hyndman-Khandakar algorithm was used for automatic ARIMA modeling [58]. This algorithm first determines the number of nonseasonal differences needed for stationarity (i.e. d in ARIMA) using repeated Kwiatkowski-Phillips-Schmidt-Shin (KPSS) tests. Then, the number of autoregressive terms and the number of lagged forecast errors (i.e. p and q in ARMIA respectively) by minimizing Akaike’s Information Criterion (AIC). The mean absolute errors (MAEs) of the ARIMA, RF, and ANN models were calculated for the 52 weeks in 2018 by the actual and the predicted numbers of dengue cases. The accuracy comparison was performed for the local (department) and national (pooled) scales.

2. An appropriate baseline model would allow the authors to calculate the relative mean absolute error which is a better measure of model performance than absolute measures such as MAE and RMSE.

*Response*: We have replaced MAE and RMSE with RMAE (relative mean absolute error). [lines 229-240]: When the comparison for an n-week-ahead prediction was conducted at the national scale, the predicted numbers of dengue cases by the 30 local RF models were additively combined and compared with the actual national values to calculate one MAE. When the comparison was implemented at the local scale, the national RF model was applied to each one of the 30 departments and then the predicted values were compared with the actual numbers of dengue cases to compute 30 individual MAEs. To improve intuitive interpretation and facilitate comparisons of one model’s predictive performance across different departments and forecasting horizons, we used the relative MAE (RMAE) to evaluate model accuracy [58]. We defined a RMAE between a ML (i.e. RF or ANN) and the baseline models at a horizon *h* as:

${RMAE}_{A,B,h}=\frac{{MAE}_{A,h}}{{MAE}_{B,h}}$	(1)

where *A* represented a ML model and *B* denoted the baseline ARIMA model.

3. Are there time lags in the dengue case data? For instance, dengue cases in Thailand had a substantial reporting lag (Reich et al., 2016. Challenges in real-time prediction of infectious disease: A case study of dengue in Thailand) and influenza cases in the US have about a 4-week reporting lag. If so, how does that affect the potential of using the RF forecasting model in real time?

*Response*: “Cases are usually reported within a week with severe cases usually being reported immediately (added on line 134).” There can be adjustments made to case counts, which typically occurs within a month maximum from report date but the adjustments are typically from probable to confirmed (and for the publicly available SIVIGILA data, the confirmed and probable are grouped together). Given this information, we believe that the reporting lag to have minimal impact on forecasting accuracy.

4. I assume that the dengue cases are the sum of probable and confirmed dengue cases from SIVIGILA. Do you know how many cases were probable and confirmed for each week? I'd like to see a supplemental figure of both plus dengue hemorrhagic fever (or severe dengue), if possible.

*Response*: Dengue cases from SIVIGILA are a mixture of probable and laboratory confirmation and unfortunately, it is not distinguished what proportion of the total reported cases belong to the different case definitions (and as stated in the Data and Method section, lines 129-133). Additionally, SIVIGILA does not provide information regarding hemorrhagic fever or severe dengue.

5. Figure 1 is okay, but if a baseline model is chosen then I would prefer to see a relative MAE figure in place of figure 2. RMSE or MAE are absolute measures that might not be comparable across locations. If one location is more variable than another, that figure may merely be displaying the difference in variability. A figure of relative MAE would show the forecasting skill by location. The sentence in the Results describing Figure 2 (on lines 245-246) is accurate, however the assertion in the discussion (lines 309-310) may change if these locations are inherently more difficult to predict for a baseline model as well.

*Response*: We have replaced RMSE and MAE by RMAE, which has been updated accordingly in Figure 2. The results description has also been updated accordingly (lines 286-290): The MAE grew for the ANN model with longer forecasting horizons compared to the local RF model. The RMAE of the ANN model obtained from the validation for 2018 was consistently smaller than that of the local RF model for each forecasting horizon. The MAE and RMAE of the national RF model were always smaller than those of the national ANN model at any forecasting horizon.

6. Is there a way to get the %IncMSEs into Table 3? The rank order is nice, but it doesn't tell us how much stronger the first variable is than the second, third, or tenth. If the lower impact variables have very low values, they could be left off in order to free up space for some numbers.

*Response*: We have included specific %IncMSE values in Table 3 (Table 5 in the revised manuscript).

7. The conclusions made by the authors are correct in stating that the national model performed better than the local model. However, this analysis doesn't tell us whether RFs are better than ARIMA or a naive baseline.

*Response*: We have included ARIMA as a baseline model and have updated our conclusions accordingly (lines 267-269): For any of the n-week-ahead (n≤12) forecasts, the national RF model more accurately predicted the counts of dengue cases than the ARIMA models, demonstrated by the smaller-than-one RMAE (Table 3).

8. The authors discuss several legitimate limitations of the study. Another limitation is that they only forecasted 2018 and that there is considerable variation between years 2014-2016 each appear to have had more incidence while 2017 had less. Whether the results are unique to 2018 or generalizable to other years remains to be seen.

*Response*: We have conducted a leave-one-season-out cross-validation in the revised manuscript and have included a new table (Table 4) (lines 284-285), which shows consistent conclusions with those obtained by the evaluation for 2018.

9. Also, the model doesn't account for the changes in population susceptibility due to the complex immunological dynamics of dengue (long-term immunity to infecting serotypes and short-term immunity to non-infecting serotypes).

*Response*: This is correct, given our approach, we do not account for population susceptibility.

10. Comment: The public health relevance is not addressed.

*Response*: We added more text in the revised manuscript regarding the public health relevance: (Conclusions, lines 357-363) Forecasting is an important warning mechanism that can help with proactive planning and response for clinical and public health services. ... These findings have important implications for dengue forecasting models in public health in terms of time savings, such as pooled data versus locally-specific models, and predictors and approaches that could help improve forecast accuracy.

11. However, the authors only compare these methods to each other and not to a simple baseline method, such as an AR-1 or an ARIMA model, which are likely to be the existing methods of choice by local public health officials. Showing how much machine learning techniques improve over the traditional standards would make for a more compelling argument.

*Response*: We included an ARIMA model as baseline.

12. Making the code and data (which are freely available) easily accessible (e.g. in a GitHub repo) would be appreciated.

*Response*: We have improved the clarity of the models in our manuscript as well as the data as a supplemental material. The senior author will create a GitHub account and post the data and code, thank you for the suggestion.

**Reviewer 2:**

**Major Comments**

1. The methods descriptions are sound and generally clear, but RF and ANN have plenty of details, and it would be nice to have more comprehensive algorithm descriptions.  Particularly important are the issues of (a) how hyperparameter tuning was performed, and (b) whether regularization was used.

*Response*: In this study, we originally used classic “non-deep” feedforward neural networks. Although we have stated that our ANN models had only one hidden layer and in many papers, ANN is particularly referred to the traditional shallow neural networks, we have realized that our expression may lead to misunderstanding for many readers because ANN in many cases is a broader term that encompasses any form of deep learning models. In the revised manuscript, we improved the ANN model from one hidden layer to three hidden layers and added “dropout” to overcome over-fitting. You will see in the new Tables 3 and 4 that errors of the improved ANN model were not larger than the local random forests model any more at all forecasting horizons.

We added more text regarding details of the RF and ANN models and especially how hyperparameters were selected in the models (lines 195-202): “Through 10-fold cross-validations, we found that the common settings for the number of variables randomly sampled as candidates at each split (i.e. the number of features divided by three) and the minimum size of terminal nodes (i.e. five) were also optimal to avoid over-fitting in our RF models [55]. The specific RF models were fitted by “randomForest” in the R statistical computing environment. We set 1000 trees to grow in the forest that is twice the default number of the package [56]. We found that further increasing the number of trees did not markedly decrease out-of-bag mean square errors of the RF models but only increased computation time.” (lines 204-216) “Artificial Neural Network (ANN) is considered a classic ML approach and to highlight the advantage of prediction accuracy of the RF models, we developed an ANN model at the national scale. The ANN was composed of one input layer, three hidden layer, and one output layer. The ANN model used ReLU as activation functions and avoided over-fitting through setting “dropouts”. Jointly considering prediction accuracy and computation time, we set “epoch” and “batch size” of the ANN models as 100 and 32 respectively. The ANN models had the same 53 predictor variables as the RF models, resulting in 53 neurons in the input layer and one neuron in the output layer. The number of neurons in the hidden layer was decreased layer by layer as the shape of an inverted pyramid. The specific number of neurons and value of dropout of a hidden layer were determined by iterative attempts until the mean absolute error (MAE) of the prediction could not be further reduced [57] (see Table 2).”

2. The results match the descriptions, and are clearly presented.  However, there needs to be evaluation on more data, e.g., by using leave-one-season-out cross-validation.  Some additional points of investigation noted in the comments might expand the contribution of this work and reinforce the points it makes.

*Response*: We have included a leave-one-season-out cross-validations in the revised manuscript and have included a new table (Table 4). (lines 241-246) “Given that dengue burden varies across different years, we conducted leave-one-season-out cross-validations to improve the robustness of our evaluation. The accuracy between the national (pooled) and local RF models as well as the national ANN model were compared. In the validations, four years of data were used to train the models and the remaining one year was used to validate the models. This procedure was iterated five times to ensure each year data were selected once for validation.”

3. Some statements seem to suggest a causal interpretation that are not warranted.  Again, as noted above, there probably needs to be evaluation on more data; I expect the current metrics on the current data to be fairly noisy.

*Response*: We have modified the wording to remove any causal interpretation and have included a cross-validation to improve the robustness of the evaluation.

4. Evaluation takes place on a single season. Even with 30 departments, this is probably not reliable. The authors describe the large differences in the different seasons from each other; it is quite reasonable to expect that relative performance of the methods could differ as well. Suggest leave-one-season-out cross-validation or a similar technique.

*Response*: We appreciate this suggestion and have added leave-one-season-out cross-validation in the revised manuscript. Please refer to previous responses for specifics about our approach or can also be found in revised manuscript in lines 241-246.

5. The performance of the methods should be compared to existing ARIMA approaches or very simple baselines, such as last-observation-carried-forward.

*Response*: We have included an ARIMA model as a baseline in the revised manuscript. Please refer to previous responses (response to the first reviewer’s first comment) for specifics about our approach or can also be found in revised manuscript in lines 220-240.

**Minor comments**

6. Why is "ANN" a "comparator"? Is it viewed as being worse a priori in this context? Is there literature to back this up? If so, does the architecture of the ANN and the amount of data in this context resemble those based on this literature? Is this applying some pre-existing approach, or not?

*Response*: In the original manuscript, the ANN was used as a baseline and constructed (shallow one-hidden layer) to match previous studies, which have been cited [line 101]. We added more text in the revised manuscript to explain why we used ANN to compare with random forests (lines 204-206): Artificial Neural Network (ANN) is considered a classic ML approach and to highlight the advantage of prediction accuracy of the RF models, we developed an ANN model at the national scale.

7. In abstract, several times throughout paper, referring to a "national" model seems a bit confusing or not very descriptive. Suggest finding an alternative description.

*Response*: We have replaced national with national pooled model.

8. "Furthermore, sociodemographic predictors are important to include to capture longer-term trends in dengue." --- This wording suggests almost a causal interpretation, but the performance analysis is not done using causal inference techniques; it would help to clarify here and any other places that suggest a causal conclusion.

*Response*: We rephrased the sentence as: “Furthermore, including sociodemographic predictors is likely to be helpful to capture longer-term trends in dengue.”

9. Line 91: missing "and"

*Response:* We have made the suggested change.

10. Line 95: suggests comparison to ARIMA

*Response*: ARIMA has been added as a baseline in the revised manuscript. Please refer to previous responses (response to the first reviewer’s first comment) for specifics about our approach or can also be found in revised manuscript in lines 220-240.

12. Line 106: parametric assumptions may be difficult to test, but I would expect that the untestable assumptions are shared by the nonparametric methods

*Response*: We reworded the sentence as (lines 97-98): “… enable the modeling of complex interactions between variables, and can fit complex models without presupposing forms (e.g. linear, exponential, and logistic) of functions [18, 19].”

13. Line 107--108: match singular/plural

*Response:* We have made the suggested change.

14. Line 109: wording may make it sound like RF is not an ML method

*Response:* We have made the suggested change and the new sentence is “Compared to the above ML methods, random forests (RF), another common ML algorithm, have shown to be more accurate …”

15. Line 113: missing comma

*Response:* We have made the suggested change.

14. Line 113--115: suggests analyzing importance of all types of variables --- performance impact of covariates / types of covariates in addition to importance rank of individual covariates.

*Response*: Meteorological factors are the most widely used covariates in forecasting dengue and there are several other predictors that have been used as it. Given the different types of modelling and evaluation approaches, it is extremely difficult to understand what predictors are the most important. We chose the predictors in our study based on the literature, what appears to be commonly strong predictors with the addition of sociodemographic predictors.

15. Line 119: Lowe et al. spatiotemporal dengue models in Brazil may qualify, but use a quite different approach

*Response*: We have slighted changed the wording of the sentence to “Universal dengue prediction models that are effective across different administrative regions remain ~~absents~~ scarce (lines 110-111).”

16. Line 136: What is the data reporting lag for probable cases? For confirmed cases? Are data revised over time? (At the end of the time series plot, is the drop down from incomplete counts to be revised?) These are important questions and the answers will almost surely indicate that performance metrics for given forecast horizons will need to be approached with some care.

*Response*: Cases are usually reported within a week with severe cases usually being reported immediately. There can be adjustments made to case counts, which typically occurs within a month maximum from report date but the adjustments are typically from probable to confirmed (and for the publicly available SIVIGILA data, the confirmed and probable are grouped together). Given this information, we believe that the reporting lag to have minimal impact on forecasting accuracy.

17. Line 140: -> "within 14 days"?

*Response*: We have made the suggested change.

18. Line 190: Nonparametric models that are not properly regularized will overfit; probably even the very simplest parametric models as well (for the stratified RF model) given the number of covariates.

*Response*: Please see our response to the reviewer’s first major comment.

19. Line 213: is there a way for RF to quantify uncertainty, and to evaluate these uncertainty estimates?

*Response*: The uncertainty of the RF predictions can be estimated by the quantile regression forests. However, this method is likely to suffer from over-fitting and is not commonly used in the practical studies. We rarely see uncertainties of RF predictions quantified in health, geographic or environmental studies and thus do not estimate uncertainty of our RF models either in this study.

20. Line 222: What about other hyperparameters, in particular: regularization parameter or number of iterations & learning rate, weight initialization, etc.? in [55], several aspects of the model are adjusted; does this work mirror that one, tune only the # of neurons, or something else? What data is used to evaluate different configurations during the tuning process?

*Response*: Please see our response to the reviewer’s first comment (major comment).

21. Line 231: is this performed using an ablation approach or a Shapley value approach? I would expect Shapley value approaches to give a better idea of contributions when certain covariates are redundant or nearly redundant with some other(s), but taken together with these redundant variables are large contributors.

*Response*: We used the standard method in random forests to calculate %IncMSE for each predictor variable and have stated clearly in our manuscript on how the %IncMSE was calculated: “Percentage of increased mean squared error indicates the increase in the mean squared error (MSE) of prediction as a result of an independent variable being randomly shuffled while maintaining the other independent variables as unchanged.”

22. Line 236: emphasizes why a leave-one-season-out or similar type of evaluation is important.

*Response*: Leave-one-season-out cross-validation has been added in the revised manuscript. We have included more text to emphasize the importance of leave-one-season-out cross-validation (lines 241-242).

23. Line 253: is there an explanation for the national/pooled RF model's better performance? Is this truly "successful transfer" or is it preventing overfitting?

*Response*: In the original manuscript, we hypothesized the reason being the pooled model performance is better than the local model, as explained in lines 312-315: “Random forests is an unsupervised tree-based regression approach requiring a relatively large training sample for the repeated splitting of the dataset into separate branches, and thus the national pooled model trained by a larger dataset had higher prediction accuracy compared to the local models.”

24. Line 254: can MAE and RMSE be put on a more interpretable scale? (Like the relative scale used for feature importance. But some similar options should be avoided if the scaling would directly emphasize instances where one particular method did well or poorly, e.g., by scaling individual errors by the error of one particular system.)

*Response*: Thank you for the comment and we used relative MAE to replace MAE in the revised manuscript (with RMSE having been removed from the revised manuscript). Please see our response to the first reviewer’s second comment for the specific revision.

25. Line 329--330: RF can also overfit, though.

*Response*: We appreciate this comment and in the revised manuscript, we re-wrote the third paragraph of the Discussion section to ensure our statement correct: “(lines 328-342) We used ANN models as comparators to our RF models. Theoretically, with ANN, more complex correlations between predictor and target variables can be discerned by deeper (i.e. more hidden layers) networks [68]. However, traditional ANN cannot handle the problem of vanishing gradient which results in the failure of improving accuracy of ANN models by adding more hidden layers. In the current study, we used the activation function of ReLU to overcome the issue of vanishing gradient, mitigated over-fitting by adding dropouts for each hidden layer, and predicted dengue cases with a three-hidden neural network. Compared with the ARIMA and local RF models, the ANN model developed by the national pooled data showed a stronger capability on forecasting dengue cases in Colombia across different forecasting horizons but performed slightly worse than the national RF model in this forecasting case study. It usually needs numerous iterative attempts to determine an optimal structure of an ANN model. By contrast, RF has some conventional settings for tuning hyperparameters (e.g. using the number of features divided by three for the number of variables at each split and five for the minimum size of terminal nodes). These default hyperparameters are usually optimal in different studies [55], which leads to an RF model relatively easy to be developed in a practical study.”

26. Line 324: shallow ANN's don't have this issue, and there are strategies to avoid these issues for deeper networks.

*Response*: Please see our response to the above comment.

27. Line 331: how are the hyperparameters set for RF? If they are tuned based on performance metrics, what training and test data is used to provide these evaluations during the tuning process? What is the exact algorithm for the RF models?  Is it really Breiman's original RF or some variant?

*Response*: Please see our response to the reviewer’s 1st and 25th comments.

28. Table 3: suggests quantifying the contribution of the AR, Year&Week, weather&vegetation, and sociodemographic categories in terms of error. (But again, care must be taken not to hint without discussion any causal conclusions; top contributors may be confounded by any number of unobserved/unincorporated quantities.)

*Response*: We have included specific %IncMSE values in revised Table 5 and have also double-checked our wording throughout the manuscript to remove any causal suggestions.

29. Line 322: -> "humans"

*Response*: We made the suggested change.

30. Line 333: this doesn't actually seem like a limitation; probably any model is going to end up with a similar phenomenon.  This does heavily suggest comparisons against last-observation-carried-forward, 1-lag or limited-lag linear autoregression, and linear autoregression augmented with week-of-season and year indicator covariates

*Response*: We added ARIMA model as a baseline in the revised manuscript to improve our comparison.

31. Line 339: this seems to be more a problem with the dimensionality than RF; we can plot the point predictions against one or two variables at a time, but issue is that it is a slice/aggregation over values for the other dimensions.

*Response*: We rephrased the sentence to be: “RF, as a non-parametric black-box approach, cannot use specific equations to quantify the relationships between the count of dengue cases and the heterogeneous predictor variables, although it is able to more flexibly and accurately capture the possibly complex non-linear and non-additive relationships among the variables (lines 348-349).”

32. Line 344: this suggest modeling changes in counts or relative changes in counts rather than the counts themselves as a target, perhaps using lagged versions as covariates.

*Response*: Thank you for the suggestion. We have included a sentence to capture this suggestion: Modeling changes in the count of dengue cases rather than the count may reduce such under-estimation errors (lines 354-355).”

**Reviewer 3**:

1. Figure 1 is hardly legible, please increase the resolution.

*Response*: We have made the suggested change.

2. The limitations such as the lack of entomological data are addressed. It would be helpful to argue why existing Aedes data was not used, was the required spatial resolution not available?

*Response*: We have clarified our reason as to why entomological data was not included in our models (it does not exist at a consistent temporal and spatial resolution in Colombia) (lines 346-347).

3. The public health implications are not sufficiently discussed.

*Response*: Thank you for this comment and we have added the public health implications in the conclusion (lines 357-363) and highlighted the time savings potential of a pooled approach (vs need to develop local models) and the approach and predictors that could be useful for disease forecasting in public health.

4. Bagging for decision tree learning hinges on the assumption of independence between observations. The dependence structures between regions for particular feature, but also between different features could be addressed by performing proper outer cross-validation for the random forest.

*Response*: We disagree that bagging for decision trees is based on the assumption of independence between observations; we have searched the literature and did not find this assumption (if there is literature on this, please do send it to us). Bagging is common practice with RFs and we believe that assessments of dependence structure are beyond the scope of our present analyses.

5. It might be helpful to compare random forest algorithms with more similar methods such as gradient descent boosting (e.g. xgboost) where carefully tuned models can potentially yield predictors with lower variance. I am not sure whether neural networks is the best suited for methodological comparison, given the limited number of features, the inherent overfitting and lack of accuracy is almost expected.

*Response*: ANN are a popular approach to disease forecasting and we continued to include ANNs in the revised manuscript as we wanted a comparison that is familiar to the readers of PLOS Neglected (versus gradient descent boosting, for example).

6. The authors show validation on the most recent part of the Dengue case count data. To confirm accuracy and robustness, it could be helpful to validate the algorithm also on other parts of the time series (e.g. years 2015-2016).

*Response:* Thank you for this suggestion, which was also a concern with the other reviewers. We have included leave-one-season-out cross-validation in the revised manuscript (lines 241-246). Please see our response to the first reviewer’s second comment for detailed changes.

7. The present study suggests the use of a broad range of environmental, climate and socio-economic data on a subnational level to accurately predict Dengue cases in Colombia. While it uses standard methods (random forests), it would be helpful to address dependence structure in the data more carefully.

*Response:* We agree that a comprehensive analysis of the dependence structure of the data is warranted and will likely be useful in informing more accurate forecasting models. We are currently working on this as a separate publication.

8. From a practical point of view, the absence of mosquito prevalence data for such predictive tasks and the lack of public health relevance should be addressed more explicitly.

*Response*: Thank you for the suggestions. We have clarified the reason as to why entomological data was not included “(lines 345-347) Including a predictor of mosquito abundance from an entomological surveillance program may reduce such time lag errors [69]. However, this type of data was not available at the national level given insufficient temporal and spatial granularity.” We added more text in the Conclusions section as to the public health relevance of the study: “(lines 357-363) Forecasting is an important warning mechanism that can help with proactive planning and response for clinical and public health services. ... These findings have important implications for dengue forecasting models in public health in terms of time savings, such as pooled data versus locally-specific models, and predictors and approaches that could help improve forecast accuracy.”

**Thank you to the reviewers for your thorough comments and suggestions.**
