# Supplementary material for: Machine learning and dengue forecasting: Comparing random forests and artificial neural networks for predicting dengue burden at national and sub-national scales in Colombia
Source: PLoS Negl Trop Dis. 2020 Sep 24;14(9):e0008056. doi: 10.1371/journal.pntd.0008056 (PMC7537891; doi:10.1371/journal.pntd.0008056)
Supplement: Attachment — Submitted filename: response_letter_Aug8.docx [file pntd.0008056.s004.docx]

Dear Dr. Choisy, Dr. Reiner, and Reviewers:

Thank you for your comments concerning our revised manuscript, “Machine learning and dengue forecasting: Comparing random forests and artificial neural networks for predicting dengue burdens at the national sub-national scale in Colombia (PNTD-D-19-02179R1).” The comments were once again extremely helpful in improving the clarity of our paper. We have provided the responses to each comment below and hope that our responses and modifications to the text are adequate.

**Reviewer 2**

1.   Line 127 -- Line 133: What type of cases are being predicted?  Suspected, confirmed, or the sum the two?

*Response*: We have clarified our text and we predicted probable and confirmed as the surveillance data includes both and does not distinguish between the two different case definitions.

2. Line 128: "confirmation" -> "confirmed cases".

*Response*: We have made the appropriate change in the text.

3. Line 132: Some readers may appreciate the additional information included in the authors' responses regarding the timeliness and nature of revisions, and the reasoning for why it may not impact predictive performance that much.  It seems more common that count surveillance systems will undergo large, biased revisions, potentially over longer time periods.

*Response*: We have included in the text to: “Cases are typically reported within a week with severe cases usually being reported immediately.”

4. Line 171: it may help to reference the discussion on line 132 here as well, or to rearrange content so that these sections are even closer to each other.

*Response*: We appreciate this suggestion but prefer to keep the two sections separate as the first paragraph is related to building the case for our approach in referring to deficiencies in previously publications and the second section is related to supporting our choice of environmental/meteorological predictors; what have been common and successfully used environmental/meteorological predictors.

5. Line 173: "Exponential smoothing approach..." is a sentence fragment.  Please specify the decay factor.

*Response*: We have clarified the sentence and also specified the decay factor: “Based on historical (2010-2017) time series data, double exponential smoothing with an additive trend was used to estimate the values for 2018. The specific exponential smoothing functions were determined by the optimal decay option in the “forecast” package for R software through minimizing the squared prediction errors.”

6. Line 222 and elsewhere: "ARMIA" -> "ARIMA"

*Response*: We have made the suggested change, thank you for this catch.

7.  Line 338: (Again, I wouldn't see this as a limitation per se; it is likely a feature of many of the best existing predictors for various epidemiological forecasting tasks.)

*Response*: Thank you for this comment and we have rephrased the sentence to “Despite the strengths of our study, our RF approach is likely to generate time lags in forecasting rapid changes in dengue, which is also a common occurrence with other forecasting approaches.”

8. Line 173: Please specify the decay factor for the exponential smoother.

*Response*: Please see our response to comment #5.

9. Line 173: "Exponential [...] data to estimate" -> "An exponential [...] data was used to estimate"

*Response*: We have made the suggested change.

10. Line 176: "local level" -> "the local level"

*Response*: We have made the suggested change.

11. Line 198: "forest that is" -> "forecast, which is"

*Response*: We have made the suggested change.

12. Line 204: "hidden layer" -> "hidden layers"

*Response*: We have made the suggested change.

13. Line 244: "therefore not suitable to conduct" -> "therefore was not suitable for conducting"

*Response*: We have made the suggested change.

14. Line 338: (Again, I don't see this as a limitation; it is likely a feature of many of the best predictors for various epidemiological forecasting tasks.)

*Response*: Please see our response to comment #7.

**Reviewer 4:**

**Major Comments**

I would like to see a more developed discussion of the pooled forecast versus the individual geographical unit forecast. There is some explanation of a larger sample size contributing to this effect, however, more explanation is needed.

*Response*: We have added more text in Discussion to further explain the pooled vs individual department forecasts: “Moreover, a RF regression model cannot yield predictions for data points beyond the scope of the training data range. Pooling data from individual departments creates a training dataset with larger ranges of variables, increasing the extrapolating capacity of the RF model.”

**Minor comments**

1. Why would education be a needed covariate, especially with SES? More

explanation of covariate choices would be helpful.

*Response*: We have clarified this in the text: “Education influences people’s knowledge and behaviours towards infectious diseases, as people with higher education more likely to adopt behaviours to reduce risks of infection compared to individuals with lower education [44]. Income also affects risk of infectious diseases, with those from higher income brackets often being less exposed and consequently, less at-risk of infection compared to individuals with lower income [45].”

2. Explanation of probabilistic statements each model can make. ARIMA can clearly

specific a probability distribution for the data, RF and ANN can use bootstrapping

to get prediction intervals, how do these compare? Forecasting seems to be

moving more towards probabilistic evaluation, including this would improve

relevance.

*Response*: It is true that the uncertainty of the RF and ANN predictions can be estimated by the quantile regression forests and the bootstrapping method, however, those methods are likely to suffer from over-fitting and is not commonly used in the practical studies. We rarely see uncertainties of RF predictions being quantified in forecasting studies and did not estimate the uncertainty of our RF models as our goal was to estimate the forecasting accuracy of our predictions. As for our ARIMA model, it was used as a baseline model and the probability distributions can easily be obtained but to match our RF and ANN models, we did not calculate the upper and lower limit values of the 95% prediction intervals.

3. Line 64: What is a department? Not a common spatial scale imho. Please define.

Response: We have clarified this in the text in the author summary.

4. Line 93: Machine learning statistical regression. Please clarify.

*Response:* We have improved our explanation in the text: “Differing from statistical modeling that forms relationships between variables based on many assumptions (e.g. independence of predictor variables, homoscedasticity, and normal distributions of errors), machine learning facilitates the inclusion of a large number of correlated variables, enable the modeling of complex interactions between variables, and can fit complex models without presupposing forms (e.g. linear, exponential, and logistic) of functions, providing a more flexible approach for disease forecasting [18, 19].”

5. Line 201: Seems like you are mixing model definition sections and model

evaluation sections. I would move up the definition of ANN and ARIMA models to

the section above and leave model evaluation as a standalone section about how

any given model will be evaluated. Maybe starting from the text on line 224

*Response*: This is a good point and we have moved all the text regarding definition of ANN and ARIMA models up to the last sub-section, and changed the title of “Development of RF models” to “Development of RF, ANN, and ARIMA models”.

6. Could be informative to describe the actual coefficient estimates of covariates for

each model?

*Response*: RF and ANN are non-parametric models and there are no coefficient estimates for each covariate.

7. Line 323: “Comparator” is strange phrasing.

*Response:* We rephrased the sentence to: “We compared our RF pooled national models to pooled national ANN models using the same predictor variables.”

8. Line 363: I don’t think you need to appeal to vanishing gradient here? This is a problem more present in recurrent neural networks. It has been well shown that a neural network with 3 layers is already sufficient to approximate any function arbitrarily well (universal approximation theorem). I think the description of the neural network methods could be improved.

*Response:* We agree and have removed the vanishing gradient comment from the last sentence of the conclusion and have also added the following sentence in the Data and Methods section to explain how the problem of vanishing gradient, that once commonly appeared in shallow artificial neural networks, was solved in the current deep neural networks: “The ANN model used ReLU as an activation function to solve the problem of a vanishing gradient and avoids over-fitting through setting “dropouts”.
